# Introduction to the special issue: “Natural Product Discovery and Development in the Genomic Era: 2021”

**DOI:** 10.1093/jimb/kuab030

**Published:** 2021-04-27

**Authors:** Ben Shen, Yi Tang, Richard H Baltz, Ramon Gonzalez

**Affiliations:** Departments of Chemistry and Molecular Medicine, and Natural Products Discovery Center at Scripps Research, The Scripps Research Institute, Jupiter, FL 33458, USA; Departments of Chemistry and Biochemistry, and Chemical and Biomolecular Engineering, University of California, Los Angeles, CA 90095, USA; CognoGen Biotechnology Consulting, 7757 Uliva Way, Sarasota, FL 34238, USA; Department of Chemical, Biological, and Materials Engineering, University of South Florida, Tampa, FL 33620, USA

In recent years, the *Journal of Industrial Microbiology and Biotechnology* (JIMB) has been publishing Special Issues devoted to advances in industrial microbiology and biotechnology, with emphasis on the core areas of the Society for Industrial Microbiology and Biotechnology (SIMB), our sponsoring society. The third international conference on “Natural Product Discovery and Development in the Genomic Era” was held on January 12–16, 2020, in San Diego, California. SIMB organized the conference, with the Korean Society for Microbiology and Biotechnology (KMB) and the Society of Actinomycetes Japan (SAJ) as co-sponsors. While this meeting is preceded by the first and second conferences on the similarly defined broad topics, held in 2015, and 2018, respectively, the origin of this series of meetings can be traced back to the Genetics and Molecular Biology of Industrial Microorganisms (GMBIM) and Biotechnology of Microbial Products (BMP) meetings. The first GMBIM meeting was held in 1976, followed by meetings in 1980, 1984, 1988, 1992, 1996, 2000, and 2004, while the first BMP meeting started in 1988, followed by meetings in 1990, 1993, 1995, 1997, 1999, 2002, and 2004. The joint GMBIM/BMP meeting held in 2004 was the last SIMB-sponsored meeting dedicated to microbial natural products. These meetings provided outstanding forums for academic and industrial scientists to discuss the latest ideas and developments on the genetics, biochemistry, and molecular biology of many industrial microorganisms, the discovery, biosynthesis, engineering, and production of microbial metabolites, and their activities and drug discovery and development.

The new series of meetings, devoted to ‘Natural Product Discovery and Development in the Genomic Era’, expands the focus on microbial metabolites to natural products of all origins—bacteria, fungi, and plants. Recent advances in microbial genomics and metagenomics have definitively revealed that the vast majority of earth's biodiversity is yet to be exploited for natural products discovery. Innovations in microbial cultivation and synthetic biology have radically expanded the chemical space of natural products and demonstrated the feasibility of engineered production of natural products from cultivated and uncultivated species in model heterologous hosts. Fundamental understanding of natural product biosynthesis has allowed rational manipulation of Nature's biosynthetic machinery for the production of both the natural products and their engineered variants. Genome mining for Nature's myriad of so-called “cryptic” natural product biosynthetic pathways has started to unveil the dark matter of natural products. Emerging strain prioritization, bioinformatics, and metabolomics technologies have revolutionized natural product dereplication, enabling structural prediction, rapid detection, and isolation of the most promising natural products at a rapid pace. Natural product discovery and development is surely undergoing a renaissance (Kalkreuter et al., 2020; Shen, 2015). Natural products remain the best sources of new drug leads and continue to play a highly significant role in the drug discovery and development process (Newman & Cragg, 2020).

The third international conference welcomed approximately 190 academia, industry, and government participants from 15 countries to discuss the most recent advances in natural product discovery and development in the genomic era. The 4 days were packed with 7 plenary sessions, featuring 44 oral presentations, and 2 poster sessions, with nearly 100 contributions, covering Natural Products of Bacterial Origin, Natural Products of Eukaryotic Origin, Novel Chemistry and Enzymology of Natural Products, Natural Product Enzymes as Biocatalysts, Enabling Technologies for Natural Products, Natural Products for New Targets and Biology, and Natural Product Drug Discovery and Development. Dr. Jon Clardy (Harvard Medical School, Boston, MA) opened the conference with a keynote lecture, titled “Molecules and Mechanisms in Gut Microbiome Studies,” setting a high standard for all the lectures to follow (Seyedsayamdost & Stallforth, 2020). Dr. William Fenical (University of California at San Diego, La Jolla, CA) delivered the banquet address, titled “Microbes from the Sea: The Challenge, the Solution and the Future,” sharing his vision into the future on ocean, biodiversity, drug discovery through the lens of a distinguished career on marine natural products over nearly a half century (Moore & Gerwick, 2015; Tomoda & McAlpine, 2020). The meeting also honors Drs. Richard Baltz and Leonard Katz as honorary co-chairs for their life-long dedication and service to SIMB and for their seminal contributions to natural product discovery and development in the genomic era. Following the special issues on the first (Baltz et al., 2016) and the second (Baltz et al., 2019) conferences, we are pleased to present the third special issue, featuring 17 reviews and original articles primarily by the invited speakers, which highlight the current advances in natural products discovery and development in the genomic era.

Enabling technologies in genomics, bioinformatics, metabolomics, and synthetic biology have fundamentally changed how to discover and produce natural products, addressing many of the challenges of traditional natural products discovery programs. Among the five excellent reviews on natural products discovery and production, Kim and co-workers provide an insightful review on WblAs, a family of global regulators presented in most *Streptomyces*, to highlight how targeted manipulation of biosynthetic gene cluster (BGC) expression could be an efficient approach to discover cryptic natural products and improve their biosynthetic titers (Nah et al., 2021). Cho and co-workers summarize the current state of the art of co-culturing actinomycetes with actinomycetes, nonactinomycetes bacteria, or fungi, as a promising approach to discover cryptic natural products by mimicking the ecological habitats to activate cryptic BGCs (Kim et al., 2021*).* Stefano Donadio and co-workers describe their recent experience in searching for new natural products by reevaluating legacy strain collections, arguing that only a small fraction of the existing microbial diversity has been explored, it is possible to find novel chemistry through a limited screening effort, and effective databases and data management however are essential to improve natural product dereplication (Zdouc et al., 2021). While these strategies focus primarily upon culturable microorganisms as a source, cyanobacteria are often slow growing, difficult for genetic engineering, or cannot be readily cultivated. Luesch, Ding and co-workers present recent advances in the heterologous production of cyanobacterial natural products in both cyanobacterial and noncyanobacterial hosts, underscoring the broader applications of synthetic biology tools in the discovery of new natural products (Dhakal et al., 2021). Finally, Zhang and co-workers review the current literature on the discovery, biosynthesis, and ecological roles of natural products from the oral microbiota, highlighting human commensal bacteria as an increasingly recognized potential source of new natural products (Barber & Zhang, 2021).

Complementary to the five reviews are six original articles showcasing the discovery of new natural products from various sources and improvement of their production titers. Bode and co-workers describe how a new deoxy-polyamine is discovered from the entomopathogenic bacterium *Xenorhabdus bovienii* by a combination of metabolomics, genomics, and expression of the engineered BGCs in selected heterologous hosts (Wenski et al., 2021). Müller and co-workers present the discovery and isolation from myxobacterial strain MSr12020, and structural elucidation of sorangipyranone, which features a unique γ-dihydropyrone scaffold and reveals new insights into polyketide biosynthesis (Okoth et al., 2021). Shen and co-workers report the discovery of the ammosesters, a new sub-family of the ammosamide family of pyrroloquinoline alkaloids, by mining the *Streptomyces uncialis* DCA2648 genome, and comparative analysis of the ammosamide and ammosester BGCs support the use of a scaffold peptide as an emerging paradigm for the biosynthesis of the pyrroloquinoline family of natural products (Luo et al., 2021). Richard Baltz highlights how the characteristic features of nonribosomal peptide synthetase (NRPS) BGCs could be exploited as molecular beacons to identify phylogenetically related BGCs encoding new natural products by BLASTp analysis of finished and draft genome sequences, as exemplified by novel lipopeptide BGCs including a new daptomycin family BGC in a draft genome of *Streptomyces sedi* (Baltz, 2021). Finally, Yoon and co-workers teach us how to further enhance clavulanic acid production titer in an industrial strain of *Streptomyces clavuligerus* by optimizing glycerol utilization, demonstrating reporter-guided mutant selection as an efficient method for further titer improvement of randomly mutagenized industrial strains (Shin et al., 2021). Shen and co-workers describe the development of submerged fermentation of engineered *Streptomyces uncialis* strains for uncialamycin production, highlighting a biotechnology platform that should greatly facilitate both fundamental studies and translational applications of uncialamycin as a promising anticancer drug lead (Hindra et al., 2021).

Natural product biosynthesis continues to serve as inspiration for new chemistry, enzymology, and metabolic pathway engineering. In a comprehensive review, Challis and co-workers detail all structurally characterized examples of the short amino acid regions at the N- and C-terminus of polyketide synthases and NPPSs, termed docking domains (DDs), and summarize efforts to utilize DDs for engineering biosynthetic pathways (Smith et al., 2021). While the use of DDs to engineer biosynthetic pathways has been undertaken with varying degrees of success, these authors caution that factors that must be maintained to successfully engineer a pathway are just beginning to be defined. In their studies of cyclic pentapeptide biosynthesis, Wakimoto and co-workers describe a structural model for the altered substrate specificity by comparing the activities of PenA and SueE, members of the penicillin binding protein-type thioesterases (PBP-type TEs) and highlight the potential use of PBP-type TEs for the cyclization of small peptides, providing a mechanistic basis for future engineering of designer biocatalysts with broadened substrate scopes (Matsuda et al., 2021).

Functionalization of C–H bonds holds enormous potential in both natural product total synthesis and structural diversity, and two thought-provoking reviews nicely articulate how biocatalysis is playing an increasing role in addressing these unmet challenges. Hyster and co-workers summarize the general mechanisms utilized in biocatalytic radical cyclizations, including P450 monooxygenases (P450s), nonheme iron- and α-ketoglutarate-dependent dioxygenases (Fe/αKGDs), and radical *S*-adenosylmethionine enzymes, underscoring the opportunities for enzymes to augment and enhance natural product synthesis using radical mechanisms (Ye et al., 2021). Hans Renata provides an insightful account on recent developments in biocatalytic methods for C–H hydroxylation of terpenoids. Renata argues that, while P450s have been preferred to date for biocatalytic oxidation in general, other oxygenase superfamilies are just as adept as the P450s for scalable and practical oxidative modifications of many small molecule scaffolds, representing a fertile area to mine for new discoveries (Renata, 2021).

No one can predict, a priori, the details of how natural products will interact with the myriad of targets that drive fundamental biological processes, and this remains true today. Two reviews highlight once again how natural products continue to teach us new biology and modes of action, potentially impacting future medicine. Abe and co-workers summarize the current state of art in the biosynthesis of the sulfonamide and sulfamate natural products, a family of organosulfur compounds that are initially only known as synthetic antibacterial drugs. Sulfonamide and sulfamate biosynthesis in actinomycetes features several distinct and unique biosynthetic machineries, exploitations of which open new opportunities for drug discovery and development (Awakawa et al., 2021). Link and co-workers review the ribosomally synthesized and posttranslationally modified peptides (RiPPs), one of the most rapidly growing superclasses of natural products in the genomic era. The rich and diverse bioactivities and their detailed modes of action of RiPPs surely will guide future efforts to prioritize genome mining of the most promising RiPPs and develop RiPPs as the next generation therapeutics (Cao et al., 2021).

The reviews and original articles included in this special issue provide only a glimpse of the innovation and progress in natural products discovery and development happening every day in the genomic era, and the state of the field is strong. While the challenges remain daunting, the opportunities are enormous. The natural products community looks forward to returning to San Diego for the fourth international conference on “Natural Product Discovery and Development in the Genomic Era,” which will be held on January 8–12, 2023.
